# Fetal supraventricular tachycardia and maternal COVID-19 vaccination: is there any relationship?

**DOI:** 10.2144/fsoa-2022-0007

**Published:** 2022-09-12

**Authors:** Wael Abdallah, Johnny B Rechdan, Razane Lakkis, Malek Nassar, Linda Daou, Nadine El Kassis, David Atallah

**Affiliations:** 1 Department of Gynecology & Obstetrics, Hôtel-Dieu de France University Hospital, Saint Joseph University, Beirut, 116-5137, Lebanon; 2 Department of Gynecology & Obstetrics, Lebanese American University Medical Center, Lebanese American University, Beirut, 116-5137, Lebanon; 3 Department of Pediatrics, Hôtel-Dieu de France University Hospital, Saint Joseph University, Beirut, 116-5137, Lebanon

**Keywords:** COVID-19 vaccine, fetal arrhythmia, pregnancy, supraventricular tachycardia

## Abstract

Fetal supraventricular tachycardia accounts for 60–80% of the fetal tachyarrhythmias with prevalence ranging from 1/1000 to 1/25 000 pregnancies. It may be secondary to fetal anomalies or maternal factors. By reviewing the literature, there is no previous article that reports fetal arrhythmia after maternal vaccination. We present herein two cases of fetal supraventricular tachycardia following the administration of the Pfizer-BioNTech COVID-19 vaccine during pregnancy. Continued safety monitoring and more longitudinal follow-up are needed to evaluate the fetal impact after maternal COVID-19 vaccination.

## Plain language summary

In the setting of the worldwide high surge of COVID-19 infection, vaccination of pregnant women is still debatable. By reviewing the literature, there is no previous article that reports fetal arrhythmia after maternal vaccination. We present herein two cases of fetal supraventricular tachycardia following the administration of the Pfizer-BioNTech COVID-19 vaccine during pregnancy. Continued safety monitoring and more longitudinal follow-up are needed to evaluate the fetal impact after maternal COVID-19 vaccination.

The normal fetal cardiac rhythm is characterized by a regular heart rate ranging between 100 and 160–180 beats per minute (bpm) with a normal 1:1 atrioventricular electromechanical relationship during each cardiac cycle [1]. Fetal supraventricular tachycardia is defined as 1:1 atrioventricular activity of the fetal heart rate exceeding 200 bpm. It accounts for 60–80% of the fetal tachyarrhythmias with prevalence ranging from 1/1000 to 1/25,000 pregnancies [2]. It is considered an important cause of fetal morbidity and mortality [1]. In fact supraventricular tachycardia is associated with non immune hydrops fetalis, which increases the risk of fetal demise, perinatal morbidities and premature delivery [3]. The basic underlying mechanism is either an automatic focus, provoking atrial contractions at a rate faster than the sinoatrial node, or a re-entry mechanism, in which there is a circular electrical current running between a fast-conduction accessory pathway, the ventricle, the atrioventricular node and the atria in either direction. Supraventricular tachycardia may be secondary to fetal anomalies such as cardiac malformation or diaphragmatic hernia [2] or maternal such as excessive caffeine consumption, smoking and illicit drug abuse. Some reports have even described a relationship between immune status abnormalities and cardiac arrhythmia [2].

On the other hand, in the setting of the worldwide high surge of COVID-19 infection, the evidence is overwhelming that the vaccines offer life-saving protection against this disease [4]. Although, pregnant women infected with SARS-COV-2 are at higher risk of severe illness than non pregnant women [5], vaccination is still debatable when it comes to pregnant women. Over 200,000 pregnant women have received COVID-19 vaccines in the world [6]. Of concern, based on our daily practice, we present two cases of fetal supraventricular tachycardia following the administration of the Pfizer-BioNTech COVID-19 vaccine during pregnancy.

## Case presentation 1

A 27-year-old woman, gravida 2 para 0 aborta 1 presented at 31^+1^ weeks for a routine antenatal visit. Her medical and obstetric history was unremarkable. Her pregnancy course was uneventful apart Pfizer-BioNTech COVID-19 vaccination at 14^+4^ weeks and 17^+4^ weeks first and second dose respectively. During her routine ultrasound, a fetal supraventricular tachycardia was incidentally identified with 220 bpm. There was no history of fever, thyrotoxicosis or excessive consumption of caffeine. Further investigations were done and showed normal maternal electrocardiogram (EKG), metabolic panel, blood count and thyroid function. The fetal sonographic scanning was normal except for a minimal pericardial effusion with no evidence of hydrops fetalis. A fetal echocardiogram was performed and revealed a structurally normal heart. The patient was admitted and digoxin 0.5 mg per os was started every 12 h. During the whole course of treatment, maternal serum digoxin, electrolytes and EKG were monitored. Fetal cardiac rate became normal after 4 days. The patient was discharged with digoxin 0.25 mg every 12 h until delivery with a plan for nonstress test weekly and continued follow-up with her obstetrician and cardiac pediatrician.

She presented at the labor and delivery room at 38^+5^ weeks with spontaneous labor. She delivered vaginally a healthy male baby with a normal heart rate. Fetal echocardiogram was normal. He started amiodarone 500 mg/m^2^/j for 8 days and then 250 mg/m^2^/j for 6 months with monitoring TSH on days 3, 10, 40 and after 6 months. He stayed in the neonatal intensive care unit for heart rate monitoring, and he was discharged after 5 days with follow-up with the cardiac pediatrician.

## Case presentation 2

A 34-year-old female, gravida 3, para 1, aborta 1 presented to the antenatal clinic for a routine visit at 29^+2^ weeks of gestation. The patient is known to have primary hypertension treated during pregnancy with methyldopa 250 mg every 12 h and nifedipine 30 mg per day. She also developed gestational diabetes, treated with insulin injections. The patient has received her first Pfizer-BioNTech COVID-19 vaccine at 27^+4^ weeks. Her obstetric history was unremarkable until this day. A fetal supraventricular tachycardia with a rate of 230 bpm was identified incidentally by routine ultrasound. No signs of hydrops fetalis, pericardial effusion or abnormal amniotic fluid index were identified. Full investigations revealed no maternal cause for the fetal arrhythmia. A fetal echocardiogram revealed a structurally normal heart.

Transplacental antiarrhythmic treatment was immediately initiated with digoxin 0.5 mg orally twice daily as a loading dose followed by a maintenance dose of 0.25 mg once daily until delivery. Bisoprolol 5 mg once daily was started instead of antihypertensive drugs. A fetal sinus rhythm was detected after 12 h of the start of the treatment with a rate of 143 bpm. Maternal serum digoxin levels, as well as electrolytes, were monitored and EKGs were done routinely. Nonstress tests were conducted on a weekly basis and close follow-ups with both her obstetrician and cardiac pediatrician were maintained.

The patient then received her second dose of the Pfizer-BioNTech COVID-19 vaccine at 31^+3^ weeks.

Preterm labor occurred at 33^+3^ weeks, 2 weeks after the administration of the second dose of the vaccine. There was no sign of maternal infection. An elective cesarean section took place under, uneventfully delivering a healthy male infant with a normal heart rate. The baby was kept under observation and arrhythmia recurred eventually at day 4 postnatally. Treatment was then launched, and the baby received amiodarone with a loading dose of 500 mg/m^2^/j per day for a total period of 8 days followed by a maintenance dose of 250 mg/m^2^/j once daily for a period of 6 months, associated with digoxin 25 mg twice daily. A fetal echocardiogram performed showed no anomalies. Rhythmic Holter is to be performed at 3 months old, and a follow-up with the pediatrician shall be kept for further evaluation.

## Discussion

Of late, the WHO recommends COVID-19 vaccination for pregnant women taking into consideration a benefit versus risk assessment [4]. On the other hand, the CDC [6] and the American College of Obstetricians and Gynecologists (ACOG) [7] recommended that pregnant individuals be vaccinated against COVID-19. In fact, pregnant women were excluded from preauthorization clinical trials, and the safety of the vaccine during pregnancy is not yet proved. A recent study published by Shimabukuro *et al.* did not show obvious safety features among vaccinated pregnant women [8]. Among 3958 pregnant vaccinated patients, we had 9.4% preterm birth, 3.2% small size for gestational age. To our knowledge, there is not a previous article in the literature that reports fetal arrhythmia after maternal vaccination, and these are the first two cases of fetal supraventricular tachycardia after maternal COVID-19 mRNA vaccine.

Vaccines can cause in general tachycardia. Park *et al.* [9] published a case of paroxysmal supraventricular tachycardia participated after each dose of pertussis vaccine in a 5-month-old child. Three reports described tachycardia as a side effect of Pfizer-BioNTech COVID-19 vaccine [10]. However, supraventricular tachycardia was associated with vaccine use independently of age and gender [11]. Thus, cardiac arrhythmia can be a side effect of the COVID-19 vaccine.

On the other hand, Gray *et al.* [12] demonstrated in their cohort study the immune transfer from vaccinated pregnant women to neonates via the placenta. Spike and RBD-specific IgG were detectable in the umbilical cord after maternal vaccination due to the cross of maternal IgG via the placenta.

The mechanism for fetal supraventricular tachycardia in our patients remains unclear. Fetal supraventricular tachycardia is defined as 1:1 atrioventricular activity of the fetal heart rate exceeding 200 beats per minute [13]. The most common cause of this arrhythmia is atrioventricular re-entry through an accessory pathway. Some reports described an association between immune status abnormalities and cardiac arrhythmia [14]. On the other hand, supraventricular tachycardia is commonly observed in patients with autoimmune diseases [15,16]. The immune transfer via the placenta and the impact of the immune response may explain the fetal supraventricular tachycardia in our cases.

Moreover, the fetal supraventricular tachycardia was detected 17 weeks after the first vaccine dose injection in the first case, while it occurred 2 weeks after the first dose in the second case. There is no evidence in the literature of the timing of the immune transfer and its durability. Further studies are necessary to identify more information about immune transfer and its impact on the fetus. Furthermore, 9.4% of patients presented preterm labor in Shimabukuro *et al.* study [8] after taking the vaccine. In our case presentation 2, the patient presented a preterm labor 2 weeks after the second dose of the vaccine. Despite the presence of diabetes and high blood pressure that are common causes of preterm birth [17], we cannot eliminate the impact of the vaccine in the induction of spontaneous preterm labor.

In conclusion, these two case presentations are representing the neonatal outcome after receiving both COVID-19 vaccine doses during pregnancy. Continued safety monitoring and more longitudinal follow-up are needed to evaluate the fetal impact after maternal COVID-19 vaccination.

**Executive summary**

**Background**

- Fetal supraventricular tachycardia accounts for 60–80% of the fetal tachyarrhythmias with prevalence ranging from 1/1000 to 1/25,000 pregnancies.
- Supraventricular tachycardia may be secondary to fetal anomalies or maternal factors.
- On the other hand, in the setting of the worldwide high surge of COVID-19 infection, the evidence is overwhelming that the vaccines offer life-saving protection against this disease.
- Vaccination is still debatable when it comes to pregnant women.
- Of concern, based on our daily practice, we present two cases of fetal supraventricular tachycardia following the administration of the Pfizer-BioNTech COVID-19 vaccine during pregnancy.

**Case presentation 1**

- A 27-year-old woman presented at 31^+1^ weeks for a routine antenatal visit when a fetal supraventricular tachycardia was incidentally identified.
- Her pregnancy course was uneventful apart Pfizer-BioNTech COVID-19 vaccination at 14^+4^ weeks and 17^+4^ weeks first and second dose respectively.
- Further investigations were done and showed no maternal or fetal etiologies.
- Fetal heart rate became sinusal after digoxin therapy.

**Cas presentation 2**

- A 34-year-old female presented to the antenatal clinic for a routine visit at 29^+2^ weeks of gestation and a fetal supraventricular tachycardia with a rate of 230 beats per minute was identified incidentally.
- She has received her first Pfizer-BioNTech COVID-19 vaccine at 27^+4^ weeks.
- Full investigations revealed no maternal cause for the fetal arrhythmia.
- Transplacental antiarrhythmic treatment based on digoxin and bisoprolol were sufficient to treat the fetal tachycardia.
- The patient then received her second dose of the Pfizer-BioNTech covid 19 vaccine at 31^+3^ weeks. Preterm birth occurred at 33^+3^ weeks.

**Discussion**

- Of late, the WHO recommends COVID-19 vaccination for pregnant women taking into consideration a benefit versus risk assessment.
- A recent study published by Shimabukuro *et al.* did not show obvious safety features among vaccinated pregnant women.
- There is no previous articles that report the occurrence of fetal arrhythmia after maternal vaccination, and these are the first two cases of fetal supraventricular tachycardia after maternal COVID-19 mRNA vaccine.
- Vaccines, in general, can cause tachycardia. Cardiac arrhythmia can be a side effect of the COVID-19 vaccine.
- Some reports described an association between immune status abnormalities and cardiac arrhythmia. The immune transfer via the placenta and the impact of the immune response may explain the fetal supraventricular tachycardia in our cases.
- Continued safety monitoring and more longitudinal follow-up are needed to evaluate the fetal impact after maternal COVID-19 vaccination.
